# Establishment of an Alphavirus-Specific Neutralization Assay to Distinguish Infections with Different Members of the Semliki Forest Complex

**DOI:** 10.3390/v11010082

**Published:** 2019-01-18

**Authors:** Lisa Henss, Constanze Yue, Joshua Kandler, Helen M. Faddy, Graham Simmons, Marcus Panning, Lia Laura Lewis-Ximenez, Sally A. Baylis, Barbara S. Schnierle

**Affiliations:** 1 Paul-Ehrlich-Institut, Department of Virology, 63225 Langen, Germany; Lisa.Henss@pei.de (L.H.); Constanze.Yue@pei.de (C.Y.); jdkandler@gmail.com (J.K.); Sally.Baylis@pei.de (S.A.B.); 2 Australian Red Cross Blood Service, Brisbane, QLD 4000, Australia; hFaddy@redcrossblood.org.au; 3 Vitalant Research Institute, San Francisco, CA 94118-4417, USA; gsimmons@vitalant.org; 4 Institute of Virology, Medical Center—University of Freiburg, Faculty of Medicine, University Freiburg, 79106 Freiburg, Germany; marcus.panning@uniklinik-freiburg.de; 5 Instituto Oswaldo Cruz, Fundaçăo Oswaldo Cruz, Rio de Janeiro, RJ 21040-900, Brazil; lialewis.fiocruz@gmail.com

**Keywords:** alphavirus, neutralization assay, lentiviral vector, pseudotyping

## Abstract

Background: Alphaviruses are transmitted by arthropod vectors and can be found worldwide. Alphaviruses of the Semliki Forest complex such as chikungunya virus (CHIKV), Mayaro virus (MAYV) or Ross River virus (RRV) cause acute febrile illness and long-lasting arthralgia in humans, which cannot be clinically discriminated from a dengue virus or Zika virus infection. Alphaviruses utilize a diverse array of mosquito vectors for transmission and spread. For instance, adaptation of CHIKV to transmission by *Aedes albopictus* has increased its spread and resulted in large outbreaks in the Indian Ocean islands. For many alphaviruses commercial diagnostic tests are not available or show cross-reactivity among alphaviruses. Climate change and globalization will increase the spread of alphaviruses and monitoring of infections is necessary and requires virus-specific methods. Method: We established an alphavirus neutralization assay in a 384-well format by using pseudotyped lentiviral vectors. Results: MAYV-specific reactivity could be discriminated from CHIKV reactivity. Human plasma from blood donors infected with RRV could be clearly identified and did not cross-react with other alphaviruses. Conclusion: This safe and easy to use multiplex assay allows the discrimination of alphavirus-specific reactivity within a single assay and has potential for epidemiological surveillance. It might also be useful for the development of a pan-alphavirus vaccine.

## 1. Introduction

Alphaviruses are predominantly transmitted by mosquitoes and cause a wide range of diseases in humans and animals. The Old-World alphaviruses, such as chikungunya virus (CHIKV), Semliki Forest virus (SFV), and the phylogenetically related Ross River virus (RRV) and Mayaro (MAYV) cause arthralgia in animals and humans. In contrast, New World viruses, such as Venezuelan (VEEV), eastern (EEEV), and western equine encephalitis viruses (WEEV) are encephalitic. SFV can cause encephalitis in rodents, but only mild symptoms in humans [1]. 

The clinical signs of the acute diseases of Old World alphaviruses are usually indistinguishable from those of infections with the flaviviruses dengue virus (DENV) or Zika virus (ZIKV), which may lead to misdiagnosis. They are characterized by a febrile illness lasting 3–7 days, rash and severe, sometimes long lasting arthralgia [2,3,4]. 

Although CHIKV, MAYV and RRV are related and cause similar disease symptoms, these viruses show different geographic distributions, dependent upon, hosts and vector utilization. MAYV was first isolated in Trinidad in 1954; since then, sporadic cases, clusters, and small epidemics of Mayaro fever have been reported from remote regions in Latin America especially the Amazon basin, but also from the city of Manaus in northern Brazil [5,6]. MAYV recently emerged in Venezuela in 2010 [7] and a case of Mayaro fever has been reported from Haiti highlighting the contemporary importance of this virus [8]. MAYV is transmitted by *Haemagogus* mosquitoes which feed on arboreal primates [9]. However, experimental transmission of MAYV by *Aedes aegypti* has been described, raising concerns that MAYV may adapt and show urban transmission such as CHIKV, which would result in serious public health problems [10]. 

In contrast, CHIKV was first described during an outbreak in southern Tanzania in 1953, but since then has been identified in nearly 60 countries worldwide [11]. CHIKV is transmitted by *Ae. aegypti* and *Ae. albopictus*. CHIKV spread was rapidly accelerated through a point mutation in its E1 protein, which improved transmission by *Ae. albopictus* mosquitoes [12]. *Ae. aegypti* is confined to the tropics and sub-tropics, however, *Ae. albopictus* also occurs in temperate and even cold temperate regions and has spread from Asia to moderate climate zones in Africa, Europe and the Americas [13]. 

RRV is endemic in Australia, Papua New Guinea and islands of the South Pacific and causes disease in humans and horses. RRV’s geographic range was thought to be limited by the distribution of its primary reservoir hosts, initially believed to be marsupials. However, recent evidence suggests that RRV circulates endemically in the Pacific Islands, where marsupials are absent [14]. In addition, RRV is a vector generalist able to be transmitted by 40 species of mosquito, which poses a serious risk of wider dissemination [15].

Currently, these alphaviruses are nearly globally distributed, and although their distribution is geographically distinct, there is a serious risk of their worldwide spread due to globalization and air traffic. Evidence for such an explosive spread of an arbovirus in a naïve population was provided by the recent infections with ZIKA in Brazil and CHIKV in Latin America [16,17]. Therefore, monitoring of alphavirus infections is necessary and requires specific diagnostic methods. Although CHIKV and O’nyong nyong virus (ONNV), MAYV and Una virus (UNV) and RRV and Sagiama virus (SAGV) are closely related, we decided to compare the viruses that cause disease in humans and that are either geographically close such as CHIKV and MAYV or currently geographically distinct such as RRV [18]. Cross-protective immunity against ONNV and induction of ONNV neutralizing antibodies has been described before for an experimental CHIKV vaccine [19]. ONNV and CHIKV are 86% identical and this indicates that some closely related alphaviruses cannot be distinguished. RRV, MAYV and CHIKV ELISAs are commercially available and some CHIKV ELISA kits detect MAYV-specific IgG, since CHIKV and MAYV cross-react in ELISAs [20,21]. RRV-specific sera also show some cross-reactivity to CHIKV [22]. To demonstrate the specificity of antibodies directed against MAYV, RRV or CHIKV, serum neutralization tests are necessary. Therefore we established a neutralization assay and characterized the neutralizing specificity of patient and blood donor plasma samples.

To develop an alphavirus neutralization assay, we extended the use of a pseudotyped lentiviral vector system [23]. Alphaviruses have a positive-sense single stranded RNA viral genome and enter cells by receptor mediated endocytosis and a subsequent pH-dependent fusion step. The early steps of infection, such as receptor binding and membrane fusion, are carried out by the viral glycoproteins, which are the major target of neutralizing antibody responses. Pseudotyping of lentiviral vectors with the glycoproteins of alphaviruses generates vectors that acquire the host range of alphaviruses. They enable studies without the need to use the native virus, which usually requires a higher laboratory biosafety level. Pseudotyped vectors are frequently used to study viral entry and to evaluate entry inhibitors [23,24,25,26,27]. CHIKV-pseudotyped lentiviral vectors have previously been described several times [23,28,29]. We expanded this vector system and established a multiplexed assay in a 384-well format for the simultaneous analysis of plasma directed against CHIKV, MAYV, and RRV. 

## 2. Materials and Methods 

### 2.1. Cell Culture

All cells used were cultured at 37 °C under 5 % CO_2_. HeLa (ATCC: CCL-2), HEK 293T (CRL-1573), NIH 3T3 (CCL-92), Huh7 (CCL-185), and A549 (CCL-185) cells were grown in Dulbecco’s modified Eagle medium (DMEM; Lonza, Verviers, Belgium). BHK 21 (CCL-10) and Jurkat (TIB-152) cells were incubated in Roswell Park Memorial Institute medium (RPMI; Biowest, Nuaille, France). All media were supplemented with 10 % foetal bovine serum (PAA, Pasching, Austria) and 5 % L-glutamine (200 mM; Lonza, Verviers, Belgium).

### 2.2. Human Samples

A human naïve plasma was obtained from a healthy volunteer in Germany. The MAYV plasma was obtained from a patient who had contracted MAYV, presenting with fever, myalgia, maculopapular rash, and polyarthralgias whilst travelling in Bolivia [30]. CHIKV antibody positive plasma samples were obtained from Puerto Rican blood donors as well as from Brazilian patients that had been clinically diagnosed with and later tested for ZIKV, DENV or CHIKV infections. Plasma samples were obtained from Brazilian patients followed at the Viral Hepatitis Ambulatory/FIOCRUZ/Rio de Janeiro following IRB approval of study amendments. The approval date was May 10, 2016 (Fiocruz IRB ID: 0142/01). RRV or BFV plasma samples were obtained from Australian blood donors and screened for the presence of RRV or Barmah Forest virus (BFV) IgG and/or IGM, following approval from the Australian Red Cross Blood Service Human Research Ethics committee. Samples labeled CHIKV (Figure 3, Figure 4 and Figure 5) were always the same sample of a Puerto Rican donor.

### 2.3. Ethics Statement

All human samples used were taken with consent of the patient for diagnostic purposes according to ethical regulations in Germany. Written informed consent was given.

### 2.4. Plasmids and DNA

The gene for the MAYV E3-E1 envelope polyprotein was synthesized by GeneArt (Thermo Fisher Scientific, Darmstadt, Germany) on the basis of the sequence of a virus isolate from the Venezuela outbreak in 2010 (accession number ALI88638, codon-optimized for expression in mammalian cells). The gene was first cut with *Pac*I and *Asc*I, blunt ended, and then cloned into the plasmid pIRES2-eGFP (digested with *Sma*I). This plasmid allows expression of genes as a bicistronic mRNA with the eGFP gene under the control of a CMV promoter. A similar strategy was used to insert the codon optimized ONNV (Gulu strain, accession number P22056) or BFV (accession number NP_054024) E3-E1 genes. The plasmids pMDLg/pRRE (encoding lentiviral Gag/Pol), pRSVrev (encoding HIV-1 Rev) [31], pCSII-Luc (encoding the lentiviral vector genome for expression of the luciferase gene) [12], pHIT-G (encoding VSV-G, Indiana-VSV strain) [32], and pIRES2-eGFP-CHIKV E3-E1 (CHIKV E3-E1 expression vector, S27-African prototype strain) [23] were used for the production of vector particles. The expression plasmids for RRV E3-E1 (T48 RRV strain) and SFV E3-E1 (A7 SFV strain) in pcDNA were a kind gift of David A. Sanders, Purdue University, West Lafayette, USA [27].

### 2.5. Lentiviral Vector Particle Production

HEK 293T cells in 10 cm dishes were cotransfected using Lipofectamine^®^ 2000 (according to the manufacturer’s protocol; Thermo Fisher, Darmstadt, Germany) with the following amounts of plasmid DNA: 10 µg pCSII-Luc, 6.5 µg pMDLg/pRRE, 2.5 µg pRSVrev, 3.5 µg pHIT-G (encoding VSV-G), and 5.3 µg pIRES2-eGFP-CHIKV, pIRES2-eGFP-MAYV E3-E1, pcDNA-RRV, pcDNA-SFV. After 24 h of incubation, the medium was replaced by 5 mL of fresh DMEM per dish. Twenty-four hours later, the supernatant containing vector particle was harvested, sterile filtered with 0.45 µm filters (Sartorius, Göttingen, Germany), and ultracentrifuged (1 h at 50,000 rpm, rotor TLA 100.3; Optima TLX Ultracentrifuge, Beckman Coulter, Krefeld, Germany) to concentrate the volume 100-fold; the particles were resuspended in DMEM and frozen in aliquots at −80 °C. VSV-G-pseudotyped vectors were frozen without concentration by ultracentrifugation.

### 2.6. Transduction of Cells with Lentiviral Vector Particles

Transduction of cells for luciferase assays was started by seeding 6000 HEK 293T cells per well in a white CELLSTAR 384-well microtiter plate (Greiner Bio-One, Frickenhausen, Germany) in a volume of 20 µL DMEM using the MultiFlo Microplate Dispenser (BioTek, Bad Friedrichshall, Germany). After 24 h incubation at 37 °C, 10 µL of luciferase encoding lentiviral vector particles (pseudotyped with VSV-G, CHIKV or MAYV Env; corresponding to a volume that would give 30,000 RLU if left untreated) plus 10 µL diluted human plasma was added to the cells using a Matrix Multichannel Equalizer Electronic Pipette (Thermo Fisher Scientific, Darmstadt, Germany). The amount of 30,000 RLU was chosen because the upper limit of the readout is 2 × 10^6^ RLU and the neutralization activity was found to be stabile for vector particles corresponding to 30,000 or 5 × 10^5^ RLU/mL. The lowest value was chosen to keep the amount of vector particles as low as possible to save reagents. Plasma dilutions ranged from 1:30 to 1:7290 (1:3 dilutions), and were mixed 1:1 with the vector particles, resulting in a final dilution of 1:60 to 1:14,580 in a volume of 20 µL. Plasma was diluted with DMEM. The vector particle–plasma mixtures were incubated in 96-U-well plates (Thermo Fisher Scientific, Darmstadt) at 4 °C for 1 h and subsequently added to the 384-well plates. From every dilution/well in the 96-well plate, 20 µL were transferred to three wells each of the 384-well plate (triplicate assay) and resulted in a total reaction volume of 40 µL. After 16 h of incubation, BriteLite (PerkinElmer, Rodgau, Germany) substrate (20 µL) was added to each well, and then incubated for 5 min at room temperature. The luciferase signal was detected with a PHERAstar FS microplate reader (BMG LABTECH, Ortenberg, Germany). 

### 2.7. Titration of Lentiviral Vector Particles

The titer of the luciferase-encoding lentiviral vectors was determined by transduction of HEK 293T with serial two-fold dilutions of vector particles in triplicates. After 16 h of incubation, the luciferase activity was measured in each well as described above. The titer was determined as luciferase units/mL. 

### 2.8. Statistical Analysis

Determination of IC_50_ values were performed using the GraphPad Prism 7.04 software (La Jolla, CA, USA) as nonlinear regression and gives as log(inhibitor) vs. response (three parameter, constrain equal to 0). IC_50_ values represent the dilution factor of the plasma sample to obtain 50% inhibition of pseudotyped vector transduction and are given without units. The mean values and standard deviations were calculated in Excel.

### 2.9. ELISA

To determine the immune status of sera against CHIKV, RRV, BFV, DENV, and ZIKV, commercially available ELISA systems were used and performed according to the manufacturer’s protocol. The following ELISA systems were used: NovaLisa Chikungunya Virus IgG qualitative Capture ELISA; EuroImmun Anti-Chikungunya virus ELISA (IgM/IgG); EuroImmun Anti-Mayaro-Virus-ELISA (IgM/IgG); Panbio Barmah Forest Virus IgG/IgM ELISA; Panbio Ross River Virus IgM/IgG ELISA; the EuroImmun Anti-Zika Virus ELISA (IgG, IgM) and EuroImmun Anti-Dengue Virus ELISA (IgG, IgM). 

## 3. Results

### 3.1. Characterization of MAYV-Pseudotyped Vector Particles

**Figure 1:**
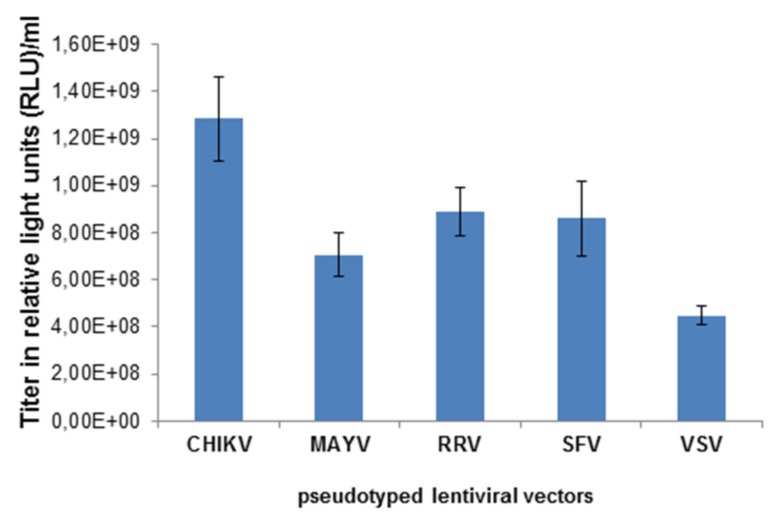
Titration of alphavirus-pseudotyped vectors. HEK 293T cells were transduced with chikungunya virus (CHIKV-), Mayaro (MAYV-), Ross River virus RRV-, Semliki Forest virus (SFV-), and vesicular stomatitis virus G-protein (VSV-G)-pseudotyped lentiviral vectors at different dilutions of the stock solution. The transduction efficiency was analyzed as relative light units (RLU) and gives the titer of the vector stock.

Lentiviral vector particles pseudotyped with VSV-G, or the MAYV, RRV, SFV or CHIKV glycoproteins (E3-E1) were generated by transfection of 293T cells with the following plasmids: a lentiviral vector genome encoding the firefly luciferase gene, the lentiviral packaging vectors and a plasmid encoding VSV-G, or the MAYV, RRV, SFV or CHIKV glycoproteins E3-E1. Two days after the transfection, cell supernatants, except those containing VSV-G pseudotypes, were concentrated by ultracentrifugation and titrated on 293T cells. Figure 1 shows the transduction of cells, determined by a whole well readout of luminescence indicated as relative light units (RLU). The vector titers were in the range of 1 × 10^8^–10^9^ RLU/mL and pseudotyping with the CHIKV E3-E1 protein was superior to RRV, SFV and MAYV. 

**Figure 2:**
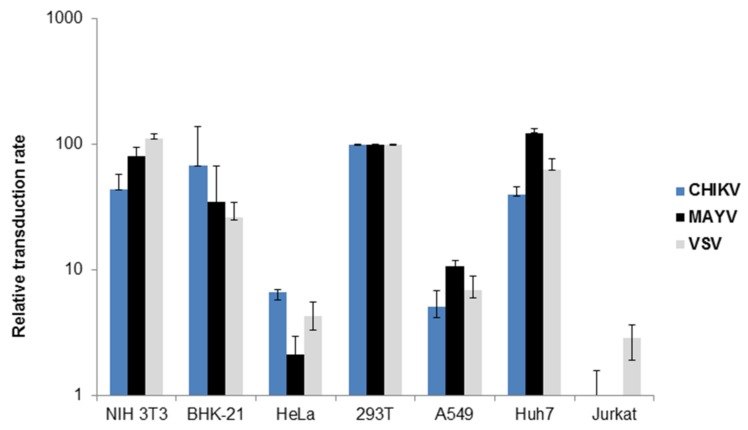
Transduction of tissue culture cells. Different tissue culture cells were transduced with VSV-G (grey), CHIKV (blue) or MAYV (black) pseudotyped vectors encoding luciferase. Transduction was analyzed as relative luciferase activity and is presented as % of transduction of HEK293T cells. The values are averages of the mean for triplicate experiments.

Since the cellular tropism of CHIKV, RRV and SFV-pseudotyped vectors has been described previously [23,27,33], we were interested in determining the cellular tropism of MAYV-pseudotyped vector particles. Tissue culture cell lines were transduced with MAYV-, CHIKV- or VSV-G-pseudotyped vectors encoding firefly luciferase. Transduction efficiency was determined again by luminescence. Lentiviral vectors pseudotyped with the VSV-G protein were used as a positive control, since they are known for their very broad cellular tropism. MAYV- and CHIKV-pseudotyped vectors transduced mouse, hamster, and human cell lines, which is in agreement with published data for CHIKV and shows, for the first time, that the MAYV cell tropism is similar to that of CHIKV [23,28] (Figure 2). As has been previously observed for CHIKV, T cells were not transduced by MAYV-pseudotyped vectors although VSV-G pseudotyped vectors were able to transduce them (Figure 2) [23,28]. A restricted ability to transduce T cells has been described for RRV- and SFV-pseudotyped vectors and therefore seems to apply to alphaviruses in general [27,33].

**Figure 3:**
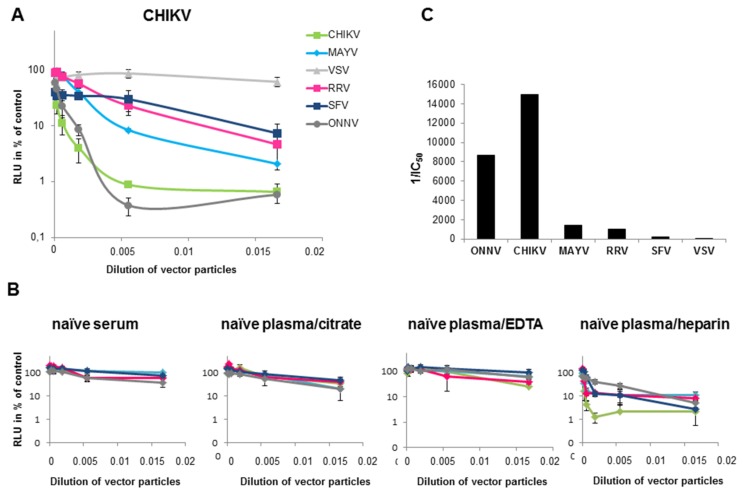
Transduction of 293T cells in the presence of human serum from a CHIKV-infected patient. HEK293T cells were transduced with CHIKV (blue), VSV-G (grey), MAYV (black), RRV (yellow), SFV (red) or O’nyong nyong virus (ONNV) (dark grey) pseudotyped vectors encoding luciferase. Transduction was analyzed as relative luciferase activity and presented as % of untreated control on a log scale. (**A**): Plasma from a CHIKV-infected patient (**B**) Serum or plasma either treated with citrate, EDTA or heparin from a healthy individual as control. The values represent mean values of triplicate measurements and IC_50_ values were calculated using the GraphPad Prism 7.04 software. (**C**) The reciprocal IC_50_ values from (A) are depicted to illustrate CHIKV-specific neutralization activity.

Next, a neutralization assay was performed with the alphavirus-pseudotyped vector particles, in order to analyze cross-reactivity of antibodies in plasma of CHIKV-infected patients. A volume of vector particles corresponding to 30,000 RLU was used. Neutralization was expressed as a percentage of the untreated transduction control. As shown in Figure 3A, the CHIKV-specific plasma had the highest neutralization activity towards CHIKV-pseudotyped vectors, followed by the specific ONNV-pseudotyped vectors. CHIKV immunization has been described before to cross-neutralize and cross-protect mice from an ONNV infection [19]. This shows the limitation of this assay, but also a general problem of discriminating closely related viruses. However, the CHIKV-specific plasma also showed partial neutralization of MAYV-, RRV- and SFV-pseudotyped vectors (Figure 3A). Based on the inhibitory concentration (IC)_50_ values (Figure 3C), the following order of neutralization was established: CHIKV > MAYV > RRV > SFV > VSV. The VSV-G-pseudotyped vectors were only marginally affected (Figure 3A,C). Serum, plasma treated with citrate or EDTA from a naïve human donor only showed non-specific inhibition of vector transduction (Figure 3B). Plasma samples that were treated with heparin should not be used for the assay, because heparin inhibits the transduction of alphavirus as well as VSV-G pseudotyped vectors [34]. Therefore VSV-G pseudotyped vectors were used as a control in further analysis. Figure 3C depicts the reciprocal IC_50_ values and illustrates the CHIKV-specific neutralization activity of the plasma from the CHIKV-infected patient. 

### 3.2. Determination of Different Alphavirus-Specific Neutralization Activities

**Figure 4:**
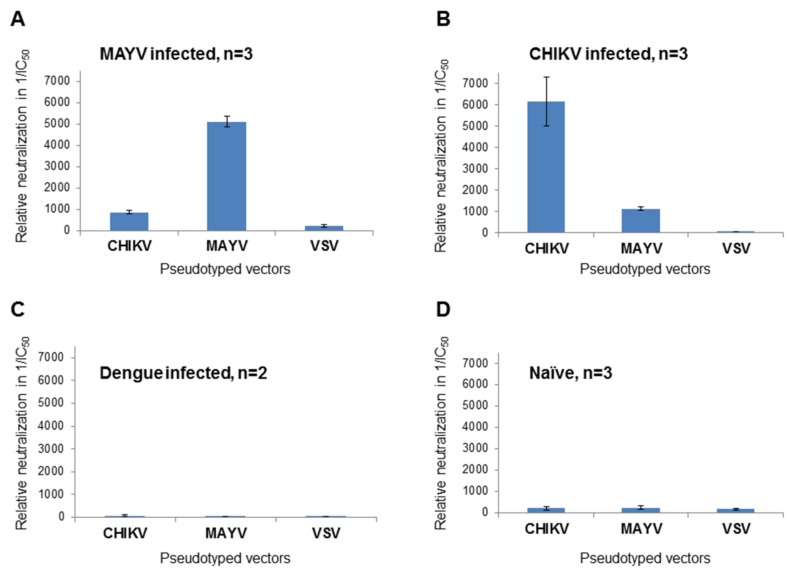
Analysis of patient samples. Plasma from (**A**) a MAYV-infected patient, (**B**) a CHIKV infected patient, (**C**) a dengue virus (DENV)-infected patient or (**D**) or a healthy control was serially diluted, incubated with CHIKV-, MAYV- or VSV-G-pseudotyped vector particles and analyzed for neutralizing activity by detection of relative luciferase activities. IC_50_ values were calculated using the GraphPad Prism 7.04 software and their reciprocal values are shown as an indication of neutralization activity. The values are the mean values of three independent (*n* = 3) experiments performed in triplicate. Only two experiments (*n* = 2) were performed with the dengue plasma.

MAYV-specific ELISAs are commercially available but cross-reactivity can be problematic [21] and an assay able to quickly identify MAYV neutralizing antibodies is still lacking. Therefore, plasma samples from MAYV-, CHIKV- and DENV-infected patients were used to perform a neutralization assay using MAYV-, CHIKV- or VSV-G-pseudotyped vectors with the intention of determining the specificity of the neutralizing antibodies present in the plasma. Vector particles and serially diluted patient plasma were added to HEK 293T cells in 384-well plates. Transduction of target cells by MAYV- or CHIKV-pseudotyped vector particles could be inhibited with the respective plasma from the infected patients. The IC_50_ values of the dose-dependent luciferase activity were calculated and their reciprocal values are depicted in Figure 4, where high values indicate neutralization. Plasma from the MAYV-infected patient showed a strong inhibition of MAYV-pseudotyped vectors and a less efficient inhibition of CHIKV-pseudotyped vectors (Figure 4A). This was reversed with plasma from the CHIKV-infected patient (Figure 4B). There was a slight cross-neutralization detectable between CHIKV and MAYV; however, specificity was indicated by the higher neutralizing activity of the specific serum (Figure 4A,B). In contrast, VSV-G pseudotyped vector particles were not inhibited by the two samples (Figure 4A,B). There was no inhibitory effect on luciferase activity after incubation of VSV-G-, MAYV- or CHIKV-pseudotyped vector particles with plasma from a DENV-infected patient or the normal healthy control (Figure 4C,D). In summary, MAYV-specific neutralizing antibodies could be discriminated from CHIKV-specific antibodies.

Furthermore, seven patient plasma samples (T1-T7) were first analyzed by ELISA for antibodies directed against CHIKV, DENV or ZIKV and the outcome is depicted in Table 1. 

**Figure 5:**
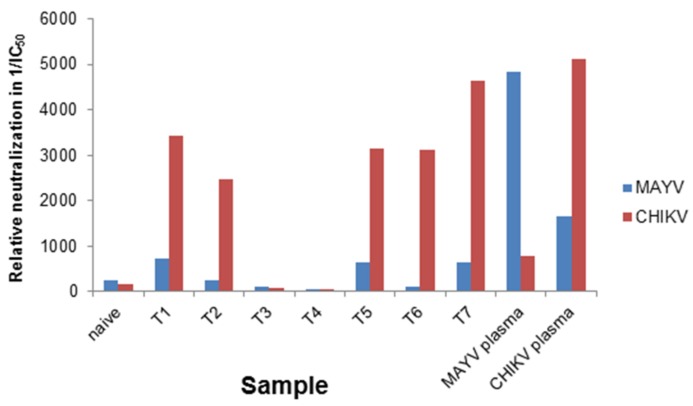
Blinded analysis of patient plasmas and graphic presentation of reciprocal IC_50_ values. The blinded samples (T1–T7) were serial diluted, incubated with CHIKV- or MAYV-pseudotyped vector particles, and evaluated for neutralizing activity by detection of relative luciferase activities as a percentage of the untreated control. IC_50_ values were calculated using the GraphPad Prism 7.04 software and the reciprocal IC_50_ value are depicted for samples analyzed with CHIKV-pseudotyped vectors (red) or MAYV-pseudotyped vectors (blue).

The samples were blinded and screened with CHIKV- and MAYV-pseudotyped vectors. The IC_50_ values of the neutralization assay were determined (Table 1), and are given as reciprocal values in Figure 5. The ratio between MAYV and CHIKV IC_50_ values is given in the last lane and indicates that a ratio above 1 determines CHIKV-specific neutralization. As a control, the MAYV-specific serum, a naïve human serum and a CHIKV-specific serum were analyzed. As illustrated in Figure 5, five of the blinded sera were CHIKV positive, indicated by reciprocal IC_50_ values above 1000 with CHIKV-pseudotyped vectors (red bars) and values below 800 for MAYV-pseudotyped vectors (blue bars). The reciprocal IC_50_ value of the MAYV-specific serum was 4847 when analyzed with MAYV-pseudotyped vectors and only 774 with CHIKV-psudotyped vectors (Figure 5). The plasma from a naïve donor and the two DENV- and/or ZIKV-positive samples had values below 100 and were correctly identified as CHIKV and MAYV negative. Although the MAYV-specific plasma gave a positive signal in a CHIKV-specific ELISA (Table 1), it could be identified here as MAYV-specific. Therefore, the reciprocal IC_50_ values can be used to determine the specificity of the sera. Very low values in both assays indicate non-specific reactivity.

To confirm the ability of the assay to determine the specificity of the sera, six plasma samples (marked with letters A–F) from Australian blood donors were first analyzed by ELISA for antibodies directed against RRV or Barmah Forest virus (BFV) virus. BFV is another Old World alphavirus only found in Australia with clinical signs similar to the other Old World alphaviruses [35]. For both viruses, commercially available ELISA assays were performed with the donor samples and the outcome is depicted in Table 2. Five samples were positive for RRV IgG and three were positive for BFV IgG by ELISA. Testing for CHIKV IgG was performed with kits from two manufacturers and showed divergent results, highlighting the issue of cross-reactivity of antibodies against different alphaviruses in diagnostic tests. In addition, the four CHIKV-positive samples were also positive in a MAYV-specific ELISA, again showing cross-reactivity.

**Figure 6:**
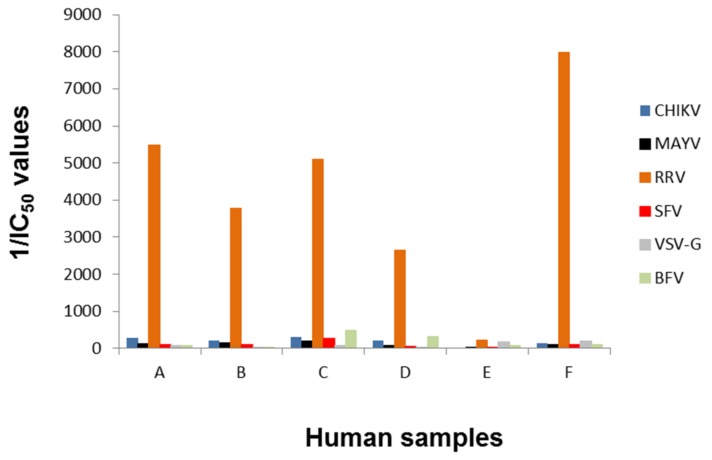
Blinded analysis of patient plasma samples. Human plasma samples (A-F) were tested blinded in serial dilution, incubated with alphavirus- and VSV-G-pseudotyped vector particles and evaluated for neutralizing activity by detection of relative luciferase activities as a percentage of the untreated control. The reciprocal IC_50_ values are depicted and were calculated using the GraphPad Prism 7.04 software, (GraphPad, San Diego, USA).

The samples were used blinded for the alphavirus neutralization assay. BFV-pseudotyped vectors were additionally included. IC_50_ values were determined and their reciprocal values are plotted in Figure 6. All samples except E were able to neutralize RRV-pseudotyped vector particles, indicated by a high reciprocal IC_50_ value (Figure 6). These data confirmed the ELISA assays, which showed that sample E was negative for RRV whereas all other samples were RRV-positive (Table 2). In contrast to the ELISA tests, no cross-reactivity with CHIKV or MAYV was observed, implying a higher specificity of the assay. Additionally, RRV could be discriminated from BFV neutralizing activity.

**Figure 7:**
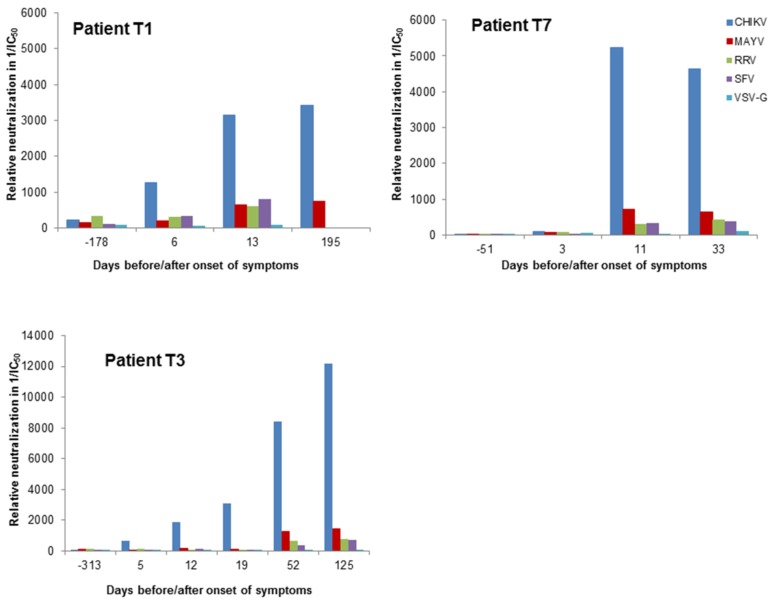
Analysis of plasma samples of three patients. Human plasma samples were tested blinded in serial dilution, incubated with alphavirus- and VSV-G-pseudotyped vector particles and evaluated for neutralizing activity by detection of relative luciferase activities as a percentage of the untreated control. The reciprocal IC_50_ values are depicted and were calculated using the GraphPad Prism 7.04 software. The onset of symptoms was on day 0.

Next, we analyzed plasma samples of three patients from Brazil, of whom one sample was included in the study in Table 1. Samples of the patients were available from time points before the CHIKV infection. Day 0 of the CHIKV infection was defined as the onset of clinical symptoms such as fever, rash and joint pain. The CHIKV infection was confirmed by ELISA (Table 1). All samples were analyzed with alphavirus-pseudotyped vectors and for control with VSV-G pseudotyped vectors. The reciprocal IC_50_ values are indicated in Figure 7. Between day 11 and 13, a drastic increase in CHIKV-specific neutralizing activity was detectable. Neutralization of MAYV-, RRV- and SFV-pseudotyped vectors increased only slightly. VSV-G-pseudotyped vectors were not neutralized by the patient samples. These data indicate again, that this assay discriminates between alphavirus-specific neutralizing antibodies. 

## 4. Discussion

Laboratory diagnosis of viral infection is usually done by detection of virus-specific antibodies in the blood, detection of virus antigens or detection of viral nucleic acids. However, for alphavirus infections, nucleic acid detection can only be used in the short viremic phase, which lasts about one week [3]. In addition, these assays are unable to provide information on the virus neutralizing activity of antibodies. To determine the neutralizing activity of antibodies or drugs, conventional neutralization assays, such as plaque reduction assays or the inhibition of cytopathogenic effects, have to be used. These assays are time consuming and rely on the use of fully infectious human pathogens. CHIKV and MAYV have to be handled in biosafety level 3 (BSL3) containment laboratories. The assays are labor intense and are difficult to automate. Pseudotyped lentiviral vector particles have the cell entry capacity of the authentic virus and can be used for the detection of neutralizing antibodies in semi-automated, high-throughput formats and at lower biosafety levels [23]. Using MAYV-pseudotyped vectors we first confirmed previous observations on the cellular tropism of alphaviruses [23,27,33]. Alphaviruses and MAYV in particular, have a broad cell tropism and could transduce all cells tested apart from T cells.

Next, we established a semi-automated neutralization assay for four different alphaviruses, using pseudotyped lentiviral vector particles and quantification by luciferase activity. This assay can be performed under BSL-2 conditions in contrast to working with the infectious virus, which requires BSL-3. The assay has a small reaction size of 40 μL, which minimizes the amount of sample material required. The automated performance of the assay, including the seeding of target cells, makes it fast and convenient. The co-screening of multiple alphavirus-pseudotyped vectors enables the identification of the specificity of antisera. In contrast, ELISA assays showed cross-reactivity among different alphaviruses (Table 1 and Table 2). The sample from a MAYV-infected person for instance was positive in a CHIKV-specific ELISA (Table 1). However, it could be clearly identified as MAYV-positive in the neutralization assay. The antigens in ELISAs are denatured proteins, while the lentiviral particles display the E3-E1 complex in its native three-dimensional structure and might be superior antigens compared to those used in ELISA assays.

The neutralization assay might be helpful for determining the global distribution of different alphaviruses particularly since commercial ELISAs are currently unavailable for some alphaviruses. However, this, as well as other assays are unable to discriminate very closely related viruses, as shown here for ONNV and CHIKV [19]. Due to globalization, expansion and adaptation to vectors and air traffic, the spread of alphaviruses is likely and has happened recently with CHIKV, which has spread rapidly in South America [36] and recent localized introductions into Europe have been reported [37]. Screening of patient samples or blood donors might help to identify outbreaks of alphavirus infections. These data might also indicate if alphavirus-specific antibodies cross-protect against another alphavirus infection or even enhance a second alphavirus infection or its disease severity [38]. Monoclonal antibodies with inhibitory activity against multiple alphaviruses have been identified and were able to protect mice in a challenge model [39]. Therefore, cross-reactive antibodies against multiple alphaviruses might be another desired goal for vaccine development and the assay could be helpful to assess this. 

The assay can easily be expanded to include more envelope proteins of alphaviruses and will enable screening of neutralizing activities against all alphaviruses within one assay. In addition, the alphavirus-neutralization assay will be helpful for alphavirus vaccine development. As CHIKV infections in immunological naïve populations can cause large epidemics which decline quickly due to the high infection rates and subsequent development of herd immunity, clinical vaccine efficacy trials are almost impossible. Neutralization titers might be suitable correlates of protection for licensure of vaccines directed against CHIKV or other alphaviruses. 

## Figures and Tables

**Table 1:** ELISA analysis and IC_50_ values of patient samples T1-T7.

Plasma	Detection by ELISA			IC_50CHIKV_	IC_50MAYV_	IC_50MAYV_/IC_50CHIKV_
	**CHIKV**	**DENV**	**ZIKA**			
**Naïve**	negative	negative	negative	6.2 × 10^−3^	4.2 × 10^−3^	0.68
**T1**	IgM (-), IgG (+)	IgM (-), IgG (+)	negative	2.9 × 10^−4^	1.3 × 10^−3^	4.68
**T2**	IgM (+), IgG (+)	IgM( -), IgG (+)	negative	4.0 × 10^−4^	4.1 × 10^−3^	10.22
**T3**	negative	IgM (-), IgG (+)	IgM (-), IgG (+)	1.1 × 10^−2^	9.1 × 10^−3^	0.80
**T4**	negative	IgG (+)	negative	2.3 × 10^−2^	2.2 × 10^−2^	0.95
**T5**	IgM (+), IgG (+)	IgM (-), IgG (+)	n.d.	3.1 × 10^−4^	1.5 × 10^−3^	4.97
**T6**	IgM (+), IgG (+)	IgM (-), IgG (+)	IgM (-), IgG (+)	3.2 × 10^−4^	1 × 10^−2^	31.58
**T7**	IgM (+), IgG (+)	n.d.	IgG (+)	2.1 × 10^−4^	1.5 × 10^−3^	7.23
**MAYV**	IgM (-), IgG (+)	n.d.	n.d.	1.2 × 10^−3^	2.0 × 10^−4^	0.15
**CHIKV**	IgM (+), IgG (+)	negative	negative	1.9 × 10^−4^	6.0 × 10^−4^	3.11

n.d. not determined.

**Table 2:** ELISA analysis and IC_50_ values of samples A–F.

	Sample					
	A	B	C	D	E	F
**RRV ELISA**	IgM (-)	IgM (-)	**IgM (+)**	IgM (-)	IgM (-)	IgM (-)
	**IgG (+)**	**IgG (+)**	**IgG (+)**	**IgG (+)**	IgG (-)	**IgG (+)**
**BFV ELISA**	IgM (-)	IgM (-)	IgM (-)	IgM (-)	IgM (-)	IgM (-)
	IgG (-)	IgG (-)	**IgG (+)**	**IgG (+)**	**IgG (+)**	IgG (-)
**CHIKV ELISA assay 1**	IgM (-)	IgM (-)	IgM (-)	IgM (-)	IgM (-)	IgM (-)
	**IgG (+)**	**IgG (+)**	**IgG (+)**	IgG (inc.)	IgG (-)	**IgG (+)**
**CHIKV ELISA assay 2**	IgM (-)	IgM (-)	IgM (-)	IgM (-)	IgM (-)	IgM (-)
	IgG (-)	IgG (-)	IgG (-)	IgG (-)	IgG (-)	IgG (-)
**MAYV ELISA**	IgM (-)	IgM (-)	IgM (+)	IgM (-)	IgM (-)	IgM (-)
	**IgG (+)**	**IgG (+)**	**IgG (+)**	IgG (-)	IgG (-)	**IgG (+)**

CHIKV Assay 1: EuroImmun Anti-Chikungunya Virus ELISA IgM/IgG; CHIKV Assay 2: NovaLisa Chikungunya Virus IgG Capture ELISA. Bold letters highlight positive reactivity of samples.
